# Finding Friends in the Crowd: Three-Dimensional Cliques of Topological Genomic Domains

**DOI:** 10.3389/fgene.2019.00602

**Published:** 2019-06-19

**Authors:** Philippe Collas, Tharvesh M. Liyakat Ali, Annaël Brunet, Thomas Germier

**Affiliations:** ^1^Department of Molecular Medicine, Faculty of Medicine, Institute of Basic Medical Sciences, University of Oslo, Oslo, Norway; ^2^Department of Immunology and Transfusion Medicine, Oslo University Hospital, Oslo, Norway

**Keywords:** 4D nucleome, genome structural modeling, Hi-C, LAD, TAD clique, TAD-TAD interaction

## Abstract

The mammalian genome is intricately folded in a three-dimensional topology believed to be important for the orchestration of gene expression regulating development, differentiation and tissue homeostasis. Important features of spatial genome conformation in the nucleus are promoter-enhancer contacts regulating gene expression within topologically-associated domains (TADs), short- and long-range interactions between TADs and associations of chromatin with nucleoli and nuclear speckles. In addition, anchoring of chromosomes to the nuclear lamina via lamina-associated domains (LADs) at the nuclear periphery is a key regulator of the radial distribution of chromatin. To what extent TADs and LADs act in concert as genomic organizers to shape the three-dimensional topology of chromatin has long remained unknown. A new study addressing this key question provides evidence of (i) preferred long-range associations between TADs forming TAD “cliques” which organize large heterochromatin domains, and (ii) stabilization of TAD cliques by LADs at the nuclear periphery after induction of terminal differentiation. Here, we review these findings, address the issue of whether TAD cliques exist in single cells and discuss the extent of cell-to-cell heterogeneity in higher-order chromatin conformation. The recent observations provide a first appreciation of changes in 4-dimensional higher-order genome topologies during differentiation.

## Introduction

Three-dimensional (3D) genome topology is important for the orchestration of gene expression governing development and tissue homeostasis. In mammalian nuclei, individual chromosomes occupy well-defined territories and adopt a radial (nuclear center-to-periphery) position which is overall conserved between cell types (Cremer and Cremer, 2010). At the nucleus scale, radial placement of chromosome territories generates topological conformations enabling a spatio-temporal regulation of processes such as DNA replication and gene expression (Bickmore and van Steensel, 2013).

A wealth of data combining high-throughput genomics, microscopy and bioinformatics has in the past decade enabled significant advancements in our understanding of spatial genome conformation at a range of scales (from gene locus to nucleus level) and resolutions (from kilobase to megabase). Comparisons between cell types and developmental studies combined with single-cell data convey an unprecedented view of common features of genome conformations and of heterogeneity in chromatin topologies at all scales. These studies also start to provide an appreciation of 4-dimensional (4D) changes in genome configuration, where the 4^th^ dimension is time. Here, we highlight recent accounts of dynamic chromatin topologies in mammalian nuclei and address the heterogeneity of higher-order chromatin conformations in light of results from ensemble data and single-cell analyses.

## A Modular 3-Dimensional Layout of the Genome

### Genomic Interactions

The combination of chromosome conformation capture techniques with high-throughput sequencing makes it possible to map 3D chromosomal interactions in entire genomes using methods such as Hi-C (Dekker et al., 2013). Hi-C is based on a chemical crosslinking of chromatin segments in close proximity (or “interacting”) in the nucleus, ligation and sequencing of these interacting segments, and mapping to a reference genome to provide a snap-shot of interacting genomic regions. The result is a matrix of interaction frequencies, often shown as a heat map, between all regions of the genome in the cell population analyzed. Hi-C data consistently show that proximal interactions, along or close to the matrix diagonal, are statistically more frequent than long-range interactions (away from the matrix diagonal) and that intrachromosomal contacts vastly dominate over contacts between chromosomes.

**Figure 1:**
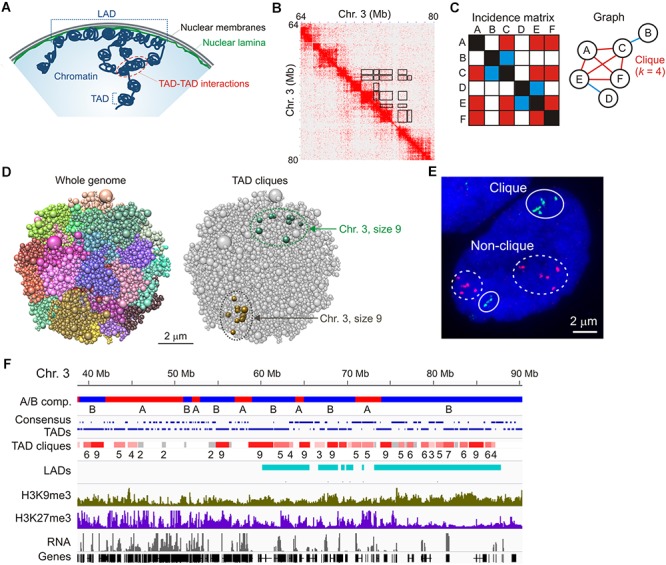
TAD cliques represent spatial assemblies of heterochromatic TADs from Hi-C data. **(A)** Higher-order chromatin topology in a mammalian nucleus, highlighting a lamina-associated domain (LAD), topologically-associated domains (TADs) and TAD-TAD interactions. **(B)** Hi-C contact matrix showing TADs along the diagonal and highlighting some of the long-range TAD-TAD interactions, away from the diagonal (black frames); shown here for a region of chromosome 3 in human adipose MSCs. **(C)** Graph representation of a clique where four vertices (or TADs; **A,C,E,F**) all interact pair-wise in the fictive incidence matrix (red cells in the matrix, red edges in the graph). Vertices B and D, respectively, interact with vertices C and E only (blue cells in the matrix, blue edges in the graph) and do not belong to the clique. The clique shown here is of size *k* = 4. **(D)** Chrom3D structural models of an MSC nucleus. Models show all chromosomes as a continuous chain of beads (TADs) labeled differently (*left*) and highlight TAD cliques of size 9 on both chromosome 3 homologs (*right*). Note the variation in TAD proximity in both modeled chromosomes. **(E)** Representative FISH image of six TADs in a clique (green probes; chromosome 1) and five TADs not in clique (red probes; chromosome 5) (see Paulsen et al., 2019 for details). Note the absence of strict physical contact between all TADs even when they belong to a clique in the Hi-C data (green probes). Also note the variation in relative TAD distributions between the two sets of homologous chromosomes. Bar, 2 μm. **(F)** Browser view of A and B compartments, consensus TADs (shown over two lines for clarity), TAD cliques with clique size (number of TADs), LADs, H3K9me3, H3K27me3 and gene expression in undifferentiated adipose MSCs. Sequence data can be accessed at NCBI GEO GSE109924 (compartments, TADs, cliques and LADs), GSM621398 (H3K9me3), GSM621420 (H3K27me3), and GSE60237 (RNA).

Analysis of Hi-C data, corroborated by microscopy approaches (Boettiger et al., 2016; Beagrie et al., 2017; Bintu et al., 2018; Szabo et al., 2018; Finn et al., 2019), suggests a segregation of the genome into multi-megabase (Mb) “open”/active A compartments and “closed”/repressed B compartments (Rao et al., 2014). Within compartments, at the 0.5–1 Mb scale, topologically associated domains (TADs) are defined as regions with a high frequency of intrachromosomal contacts, whereas contacts are much less frequent between adjacent TADs (Dixon et al., 2012; Nora et al., 2012; Figure 1A). Within TADs, the number and nature of contacts can vary, partially specifying gene regulatory interactions (Kragesteen et al., 2018). Along the linear (1-dimensional) genome, TAD borders are overall conserved between cell types (Rao et al., 2014) and disrupting or weakening of TAD borders, for example by mutations in DNA binding motifs for structural proteins, can cause disease (Guo et al., 2015; Lupianez et al., 2015, 2016; Ren and Dixon, 2015) or be oncogenic (Hnisz et al., 2016; Valton and Dekker, 2016). Nevertheless, interactions between TADs (Olivares-Chauvet et al., 2016; Beagrie et al., 2017; Niskanen et al., 2018; Quinodoz et al., 2018; Szabo et al., 2018) and positioning of TADs in the nucleus space (Li et al., 2015; Paulsen et al., 2019) can vary between cells, conveying the idea that spatial genome topology displays cell-to-cell heterogeneity in a population and is therefore not static.

### The Nuclear Lamina Provides Anchors for Chromatin at the Nuclear Periphery

3D genome conformation is also under the influence of interactions of chromosomes with the nuclear envelope, at the nuclear periphery (Zuleger et al., 2013; Lund et al., 2014; Czapiewski et al., 2016; van Steensel and Belmont, 2017; Buchwalter et al., 2019). Subjacent to the nuclear membranes lays the nuclear lamina, a meshwork of intermediate filament proteins called lamins; these consist of lamins A and C (also referred to as lamins A/C or indiscriminately here as “lamin A” because they are splice variants of the *LMNA* gene) and lamins B1 and B2, products of the *LMNB1* and *LMNB2* genes (de Leeuw et al., 2018). Genomics and microscopy studies show that nuclear lamins interact with chromatin via lamina-associated domains (LADs) (Guelen et al., 2008; Buchwalter et al., 2019; Figure 1A). While lamin B1-chromatin interactions (lamin B1 LADs) are predominantly detected at the nuclear periphery, lamin A-associated regions have been shown to occur both at the nuclear periphery (lamin A LADs) and in the nuclear interior (Kind et al., 2013; Lund et al., 2013; Lund et al., 2015), in agreement with the existence of a nucleoplasmic pool of chromatin-bound lamin A (Naetar et al., 2017). Overall, peripheral LADs are gene-poor, heterochromatic and transcriptionally silent, however intranuclear lamin A-associated regions tend to be more gene-rich and euchromatic, and contain expressed genes (Lund et al., 2015; Gesson et al., 2016). This supports the view that nuclear lamin A in particular is able to associate with genomic regions harboring distinct chromatin features. This property may underline the broad impact of lamin A on the radial positioning of chromatin (Solovei et al., 2013; Thanisch et al., 2017), higher-order and locus-level chromatin conformation (Cesarini et al., 2015; Rønningen et al., 2015; Gesson et al., 2016; Oldenburg et al., 2017; Paulsen et al., 2017; Briand et al., 2018; Grigoryan et al., 2018; Forsberg et al., 2019; Ulianov et al., 2019) and chromatin mobility (Bronshtein et al., 2015, 2016; Vivante et al., 2018). It is important to mention, however, that although A-type lamins are able to bind DNA and nucleosomes *in vitro* (as do B-type lamins) (Bruston et al., 2010), they are not sufficient to anchor heterochromatin at the nuclear periphery, and rather do so via lamin-associated protein complexes containing integral proteins of the inner nuclear membrane (Solovei et al., 2013; Buchwalter et al., 2019).

Mapping of LADs during cell differentiation suggests that a proportion of lamin-chromatin interactions are developmentally regulated (Peric-Hupkes et al., 2010; Lund et al., 2013; Rønningen et al., 2015; Robson et al., 2016, 2017). In mesenchymal stem cells (MSCs) from human adipose tissue, promoters of genes that control adipogenesis and that are bound to lamin A in undifferentiated cells, have been shown to dissociate from lamin A after induction of adipogenesis (while genes regulating other lineages do not), in a manner that could prime these genes for activation (Lund et al., 2013). Variable lamin A-chromatin interactions are detectable not only at individual loci but also encompass entire regions (Rønningen et al., 2015; Gesson et al., 2016). An additional level of complexity of chromatin association with nuclear lamins are occurrences of exchangeable interactions of chromatin with lamins A and B1 in an *in vitro* steatosis cell model (Forsberg et al., 2019). Computational models of 3D genome structure corroborated by fluorescence *in situ* hybridization (FISH) illustrate, based on these lamin-chromatin interaction data, a radial repositioning of TADs as a function of whether or not they contain lamin B1 LADs (Forsberg et al., 2019). Dynamic interactions of chromatin with the nuclear lamina, and lamina-associated protein complexes, thus provide a means of radially (re)positioning chromatin in the nucleus (Reddy et al., 2008; Kind et al., 2013; Solovei et al., 2013; Harr et al., 2015; Kind et al., 2015).

How TADs and LADs as genomic organizers together orchestrate spatial genome topology has recently been investigated in a controlled differentiation system (Paulsen et al., 2019). The findings, discussed below, provide evidence of multiple long-range TAD-TAD interactions which, together with associations with the nuclear lamina through LADs, contribute to shaping the 4-dimensional genome during terminal differentiation.

## A Get-Together of TADs Into Cliques

### How Are TAD Cliques Recognizable?

Hi-C matrices typically reveal interactions within TADs, between linearly consecutive TADs (along the matrix diagonal), and between linearly non-contiguous TADs – that is, away from the matrix diagonal (Figure 1B, exemplified in boxed areas). Such long-range TAD-TAD interactions involve only two TADs (TAD pairs) or multiple TADs. In addition, when multiple TADs interact in the Hi-C data, all TADs can interact with one another in this “network,” or only a subset of TADs does. Identifying multiple TAD-TAD interactions in a Hi-C matrix can therefore constitute a real challenge. One way to overcome this is to turn to the mathematical area of graph theory and cliques. In graph theory, a clique is a subset of *k* vertices (or nodes) which are all connected pair-wise by an edge (Figure 1C). In a recent interrogation of changes in long-range TAD-TAD interactions during differentiation, we defined a “TAD clique” as a subset of *k* TADs (with *k* ≥ 3) which are fully connected – that is, which all interact pair-wise in the Hi-C data (Paulsen et al., 2019; Figure 1C).

A key step in the identification of TAD cliques is mapping statistically significant pair-wise TAD-TAD interactions. This has been achieved using a non-central hypergeometric distribution to calculate the probability of observing a given number of Hi-C contacts dependent on the number of contacts involved between the two TADs, the total number of contacts the two TADs are involved in, and the genomic distance between the TADs (Paulsen et al., 2018). A *P*-value is then computed to identify statistically significant contacts – i.e., contacts that occur more frequently than what would be expected by chance. TAD cliques are subsequently identified by representing all significant inter-TAD contacts as a graph (Figure 1C) and searching for maximal clique sizes (Paulsen et al., 2019). Using this approach, we found that TAD cliques represent a prominent feature of higher-order genome organization: from ∼15,000 significant pair-wise inter-TAD contacts mapped in human adipose MSCs, we found more than 3,000 cliques of 3–11 TADs which altogether make up ∼50% of the genome.

Three-dimensional structural models of the genome (Paulsen et al., 2017) predict long-range inter-TAD interactions for TADs in cliques that are more frequent than that of TADs in a randomized topology (Figure 1D; Paulsen et al., 2019). Dual-color FISH using probes against TADs in cliques and outside cliques supports the modeling predictions and illustrates that TADs in cliques can form close associations, as exemplified in Figure 1E. However, as discussed later, variations in how physically close to one another TADs in a clique are, demonstrate the heterogeneity in chromatin configurations between cells and challenges the interpretation of ensemble Hi-C data.

### TAD Cliques Form Higher-Order Chromatin Assemblies Identifiable in the Hi-C Data

TAD cliques are enriched in B compartments and accordingly, genes in cliques are overall repressed or lowly expressed (Figure 1F). TAD cliques are enriched in trimethylated histone H3 lysine 9 (H3K9me3) usually throughout the TADs, and to a greater extent than in the Polycomb mark H3K27me3 (Figure 1F). Thus TAD cliques exhibit characteristics of constitutive heterochromatin and may harbor Polycomb domains. H3K9me3/H3K27me3 enrichment profiles in TAD cliques suggest that they represent a subtype of B compartment previously unrecognized (Rao et al., 2014), containing H3K9me3 with variegated H3K27me3 and, as discussed later in this article, variable LADs.

TAD cliques are also found in A compartments, yet in lower proportions than in B compartments (Paulsen et al., 2019). Intriguingly, TAD cliques in A compartments include active genes interspersed with H3K27me3-marked genes, but overall harbor no LADs (Figure 1F). In undifferentiated cells, such cliques may represent associations of facultative heterochromatin containing genes that can be activated during differentiation.

### TAD Cliques Represent Dynamic Topological Assemblies

TAD cliques are not static entities and following their fate during differentiation reveals the dynamics of higher-order chromatin topologies. Supporting this idea, using an alluvial graph representation, TAD cliques have been shown to expand or shrink during adipose differentiation, by gaining or losing TADs, and some cliques also exhibit adipose versus neuronal lineage-specificity (Paulsen et al., 2019). In line with the repressed nature of TAD cliques, clique expansion is associated with downregulation of expression of genes within the clique, and conversely, down-sizing of a clique coincides with upregulation of gene expression. Changes in clique size do not correspond to changes in B compartment size or to A/B compartment switching, suggesting that TAD clique dynamics constitutes yet another level of higher-order chromatin conformation changes.

Temporal changes in inter-TAD contacts characterize not only mesenchymal and embryonic stem cell differentiation (Bonev et al., 2017; Paulsen et al., 2019), but also dedifferentiation, as shown during the reprogramming of mouse B cells toward pluripotency (Stadhouders et al., 2018). Remarkably, during cell reprogramming, a striking reduction in the number of TAD cliques detected in B cells (Paulsen et al., 2019) likely reflects a loosening of higher-order chromatin structure as cells acquire a pluripotent state. Inter-TAD contacts also appear to be prone to environmental conditions. The heat shock response in *Drosophila* cells is topologically manifested by a decrease in contacts within TADs (perhaps reflecting gene expression changes) and an increase in long-range inter-TAD interactions (Li et al., 2015). This implies a spatial rearrangement of TADs and a large-scale reorganization of chromatin which may be important for gene silencing after temperature stress. How long-range TAD-TAD interactions are promoted in this system remains unknown but could implicate a decrease in the strength of TAD borders (Li et al., 2015). These studies exemplify how dynamic long-range interactions between topological domains, such as a gain or loss of TADs in cliques, emerge as functionally important processes shaping the 4D nucleome.

## TAD Cliques and Other Long-Range Associations Between TADs

### Cliques and SPRITE Hubs

Chromatin is anchored to intranuclear bodies, including nucleoli (Nemeth et al., 2010) and nuclear speckles (Baudement et al., 2018; Chen et al., 2018). The split-pool recognition of interactions by tag extension (SPRITE) method was developed to detect higher-order multi-way chromosomal interactions (Quinodoz et al., 2018). Over 300,000 so-called SPRITE clusters of 3–14 “*k*-mers” (or associations) have been reported. These associations were interpreted to form “chromosomal hubs” arising from long-range interactions including either gene-dense, active and RNA-polymerase II-marked regions at nuclear speckles, or inactive centromere-proximal regions at the nucleolus (Quinodoz et al., 2018). Since unlike Hi-C, SPRITE does not depend on proximity ligation, the method enables detection of genomic interactions over longer distances than those detectable by Hi-C (Quinodoz et al., 2018). The heterochromatic nature of cliques and of nucleolus-associated domains (Nemeth et al., 2010; Sen Gupta and Sengupta, 2017) raises the possibility that a fraction of repressed SPRITE clusters could reside in TAD cliques or encompass several cliques at the periphery of nucleoli.

### TAD Cliques and Long-Range Inter-TAD Interactions in Other Systems

Heterochromatic TAD cliques resemble H3K9me3-rich “TAD hubs” reported in B compartments as a result of long-range inter-TAD contacts in the Hi-C data in endothelial cells, and similarly to cliques, these “hubs” are enriched in LADs (Niskanen et al., 2018). Interestingly, analyses of TAD cliques and of the “hubs” of Niskanen et al. concur in that despite the clique rearrangements discussed above, most chromatin domains seem to fall within a pre-established overarching conformation (such as TAD cliques or absence thereof) that is overall maintained during terminal differentiation (Niskanen et al., 2018; Paulsen et al., 2019).

TAD assemblies have also been reported in *Drosophila* using Hi-C and 3D FISH. The data interestingly reveal higher-order dynamic interactions between TADs, where repressed TADs are organized as a succession of “nanocompartments” interspersed by active regions (Szabo et al., 2018). Some of these nanocompartments involve linearly non-adjacent TADs (as suggested by FISH and inferences from 3D models of these configurations), supporting the idea of TAD cliques. The TAD assemblies of Szabo et al. also resemble TAD cliques in A compartments harboring H3K27me3 and similarly to these particular cliques, they seldom occur (Szabo et al., 2018). The Paulsen and Szabo studies also concur in that changes in inter-TAD interactions reflect discrete chromosomal contacts and not a fusion or splitting of TADs.

Other studies also provide evidence of inter-TAD interactions, but properties of these interactions distinguish them from TAD cliques. (i) “Meta-TADs” have been reported as interactions between multiple neighboring TADs and thus do not encompass strictly long-range TAD-TAD interactions that define cliques. Meta-TADs are enriched in H3K27me3 and RNA polymerase II (Fraser et al., 2015) but are devoid of H3K9me3, which again segregates them from TAD cliques. (ii) A variation of Hi-C using “chromosome walks” (C-walks) captures associations between two to four TADs, the occurrence of which is enhanced by Polycomb group proteins (Olivares-Chauvet et al., 2016). Interestingly however, the C-walk data favor a view of pair-wise TAD-TAD contacts over a hub-like topology, and random associations between active loci rather than a regulated process. (iii) Genome architecture mapping, a method that measures genomic contacts based on the sequencing a large number of thin slices through nuclei, has been shown to identify three-way TAD interactions (Beagrie et al., 2017). These multivalent interactions regroup active genes and enhancers (Beagrie et al., 2017) and may constitute supra-TAD gene regulation units.

## When TAD Cliques Become Peripheral Matter

**Figure 2:**
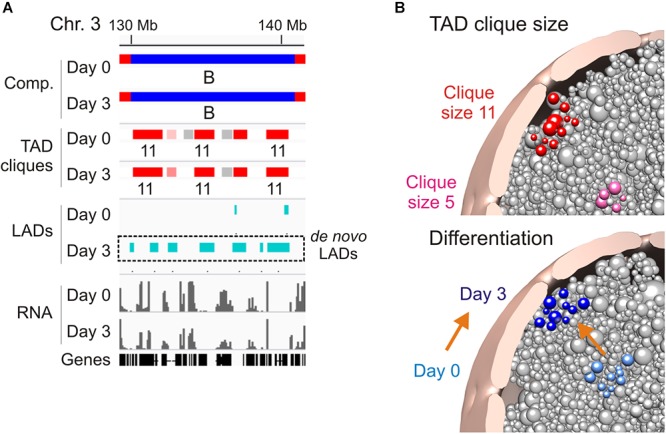
Association of TAD cliques with the nuclear periphery. **(A)** Browser view showing *de novo* LADs appearing in pre-existing TAD cliques in a B compartment on day 3 of adipose differentiation. Sequence data can be accessed at NCBI GEO GSE109924 and GSE60237. **(B)** Radial positioning of TAD cliques in the nucleus: model views. *Top*, differential preferred radial position of a small (pink) and a large (red) TAD clique. *Bottom*, differentiation repositions a TAD clique toward the nuclear periphery; orange arrow symbolizes differentiation.

A feature of TAD cliques in human and mouse cells is their enrichment in LADs, however, this relationship seems to depend on clique size and cell state (Paulsen et al., 2019). Accordingly, the proportion of linear clique coverage by LADs increases with clique size (up to 50% in large cliques), and adipogenic induction coincides with an increase in the LAD content of cliques irrespectively of clique size. This implies that large cliques tend to associate with the nuclear lamina and that this association is exacerbated in terminally differentiated cells. Nevertheless, lamina association appears to be dispensable for TAD clique formation because many cliques exist in the absence of LADs (Figure 1F; see region 40–60 Mb in chromosome 3), and there are several instances of LADs emerging within already established cliques during adipose differentiation (Figure 2A; *de novo* LADs). Interestingly, nuclear lamina anchoring of TADs in cliques may further compact chromatin in these TADs (Ulianov et al., 2019).

Three-dimensional genome structural models corroborate these features and predict a nuclear peripheral localization of TAD cliques in relation to clique size, with larger cliques more frequently found at the nuclear periphery, and differentiation (Paulsen et al., 2019; Figure 2B). Given their heterochromatic nature, it is reasonable to speculate that TAD cliques may strengthen a repressive state of gene expression by stabilizing peripheral heterochromatin at the nuclear lamina. To achieve this, our data argue that only a subset of TADs would be sufficient to anchor a clique at the nuclear lamina since within a clique containing LADs, not all TADs necessarily harbor LADs. Thus, a peripheral localization of TADs in a clique may not directly require LADs if this localization implicates LADs in neighboring TADs. The clique concept further argues that these neighboring TADs need not be linearly contiguous as long as they remain spatially close in a 3D environment.

## Are There TAD Cliques in Single Cells?

TAD cliques are currently identified from Hi-C data generated from millions of cells, so Hi-C data reflect averages of chromosomal interactions across a cell population and do not reflect genomic interactions in individual cells. This knowledge gap has prompted the advent of single-cell Hi-C as a technical *tour-de-force* (Nagano et al., 2013, 2017; Flyamer et al., 2017; Stevens et al., 2017). Single-cell Hi-C captures snapshots of chromosomal interactions in individual cells, and although contacts are sparser than in ensemble Hi-C matrices, it is possible to detect statistically significant pair-wise TAD-TAD contacts (Paulsen et al., 2019). Nevertheless, this sparsity of contacts makes identification of TAD cliques virtually impossible.

To circumvent this problem, we have proposed a five-step proxy strategy which enables an estimation of TAD-TAD contacts within projected TAD cliques identified from ensemble Hi-C data:

• Determine significant pair-wise TAD-TAD interactions in single-cell Hi-C contact matrices;
• Map TAD cliques in ensemble Hi-C data for the same cell type;
• Project these cliques onto individual single-cell Hi-C matrices;
• Calculate TAD contact frequencies within the projected cliques and outside the cliques;
• Calculate TAD contact densities in the projected cliques.

Using this approach, we found in mouse embryonic stem cells an enrichment of TAD-TAD interactions in projected cliques compared to randomized controls (Paulsen et al., 2019). Further, most single cells analyzed display clique-like TAD assemblies with at least 50% TAD connectivity within them (that is, with more than 50% of TADs connected pair-wise within the projected cliques in the single-cell Hi-C data). Thus, although this does not demonstrate the existence of TAD cliques in single cells, the subsets of TADs may display statistically significant long-range associations also in single-cell Hi-C data.

## Heterogeneity in Higher-Order Chromatin Topologies

### FISH and Hi-C: Variations in Locus Proximities

At the level of the nucleus, pair-wise TAD-TAD contacts can be highly variable between cells in a population. Single-cell Hi-C data show variability in the number and genomic coordinates of chromosomal interactions (Nagano et al., 2013, 2017; Flyamer et al., 2017; Stevens et al., 2017) and in significant inter-TAD contacts. This is also seen in the number and nature of TADs involved in projected TAD cliques using the approach outlined above. FISH analysis corroborates the Hi-C data and reveals heterogeneity in spatial TAD proximity (Szabo et al., 2018; Finn et al., 2019; Paulsen et al., 2019; see Figure 1E). Therefore, sets of TADs may preferably be in proximity at the single-cell level but not necessarily in physical contact in all cells. Stochastic interactions within this proximal neighborhood, which has led to the view of “stochastic clusters” (Flyamer et al., 2017), can still be statistically more frequent than stochastic interactions between TADs in a spatial random configuration.

Heterogeneity in FISH configurations is also detected for homotypic TADs that exhibit “statistical preference” (Boettiger et al., 2016; Szabo et al., 2018). Corroborating this view, C-walks favor the idea of stochastic TAD-TAD interactions rather than functional interaction hubs (Olivares-Chauvet et al., 2016). Similarly, analysis of chromosome conformation by high-throughput FISH shows that even neighboring TADs do not necessarily cluster, and concurs in that cell populations display a wide array of genome configurations (Finn et al., 2019). Some of this variation can be caused by inter-allelic variation within single nuclei (Oldenburg et al., 2017; Finn et al., 2019; Paulsen et al., 2019; see also Figure 1D,E), which adds to the challenge of interpreting Hi-C data in the absence of sufficient polymorphism. These observations altogether illustrate the variegated higher-order topologies of chromatin between cells and between homologous chromosomes in a given cell.

### Cell-to-Cell Variability in Genome Conformation Estimated From Structural 3D Models of the Genome

With recent developments in computational approaches to model genome structure in 3D, it is now possible to make powerful estimations of the spatial arrangement of chromatin domains, including their radial positioning and their spatial proximity (Paulsen et al., 2018). Several frameworks and pipelines can model 3D genome structures (Imakaev et al., 2012; Dekker et al., 2013; Serra et al., 2015; Szalaj et al., 2016; Tjong et al., 2016; Li et al., 2017; Paulsen et al., 2017), and some can incorporate locus positional constraints in the modeled nuclei. Integrated Modeling Platform (IMP) is a framework initially developed to model 3D protein structure, and can in principle integrate spatial restraints for chromatin (Kalhor et al., 2011; Bau and Marti-Renom, 2012; Russel et al., 2012; Tjong et al., 2016; Li et al., 2017). Other methods enable integration of non-Hi-C data in the modeling, such as nucleolus constraints and centromere position in yeast (Duan et al., 2010), or interactions with the nuclear lamina (LADs) (Li et al., 2017; Paulsen et al., 2017). We have recently introduced Chrom3D (Paulsen et al., 2017, 2018), a platform designed to incorporate Hi-C and lamin ChIP-seq data as positional constraints for TADs (or any other genomic unit); these provide respective information on inter-domain interactions and on the radial positioning of loci in the modeled nuclei (Briand et al., 2018; Forsberg et al., 2019). Analyses of 3D genome models enable statistically robust estimates of variations in 3D genome structures between cells in the population under study (Paulsen et al., 2017). Using deconvolution techniques, IMP-based approaches estimate an ensemble of structures as part of a single simulation (Dai et al., 2016; Tjong et al., 2016; Li et al., 2017). In contrast, Chrom3D generates a single structure per simulation, and hundreds of simulations allows for statistical estimates of the position of domains across a large number of models (Paulsen et al., 2017).

The modeling exercises predict that heterogeneity in 3D genome structures exist between cells in a population, both in terms of spatial proximity of given domains (Paulsen et al., 2019) and in their positioning relative to the nuclear periphery (Dai et al., 2016; Tjong et al., 2016; Li et al., 2017; Paulsen et al., 2017; Briand et al., 2018; Forsberg et al., 2019). This variation, visualized by FISH (Kind et al., 2015; Paulsen et al., 2017; Briand et al., 2018; Finn et al., 2019; Forsberg et al., 2019), emerges as a significant factor impacting higher-order genome topologies. Chrom3D modeling reveals that TADs in cliques show closer proximity than TADs outside cliques in a control random configuration (Paulsen et al., 2019). Importantly however, TADs in cliques (as seen in Hi-C data) are rarely, if at all, all closely associated in a given modeled nucleus (or in FISH experiments) (see Figure 1D,E), in line with the interpretation of ensemble versus single-cell observations. At present, we do not know whether population-based modeling provides more information than statistical estimates from multiple simulations. Despite validations of predicted 3D genome structures by FISH (Tjong et al., 2016; Li et al., 2017; Paulsen et al., 2017, 2019; Forsberg et al., 2019), more work is required to determine whether variations in chromatin topologies can also be reliably predicted by computational modeling.

## Concluding Remarks and Future Directions

A role of TADs and LADs in spatially organizing the genome has been established through their respective purpose in confining gene regulatory interactions and anchoring chromatin at the nuclear periphery. The nuclear lamina has been shown to anchor subsets of TADs at the nuclear envelope through LADs in a differentiation-dependent manner (Robson et al., 2016; Paulsen et al., 2019), providing a radial relationship between these genomic organizers. The recent results presented here highlight a new level of 4D genome organization involving long-range TAD-TAD associations into TAD cliques and a radial positioning of cliques related to their LAD content. Of note, TAD cliques can exist in the nuclear interior without LADs, making LADs unlikely to be required for cliques to assemble. LADs may, however, be necessary to stabilize cliques containing long-term repressed developmental genes at the nuclear periphery. Following the dynamics and spatial (re)positioning of TAD cliques during development, lineage commitment and terminal differentiation, in relation to the evolution of epigenetic components, including chromatin accessibility and DNA methylation, is expected to provide new insights on higher-order genome topologies in a 4-dimensional context.

A key question remains of how TAD cliques are formed and disassembled. TAD cliques in B compartments are primarily heterochromatic. So mechanistically, proteins promoting the formation or spreading of heterochromatin, such as heterochromatin protein 1 (HP1) isoforms (CBX1, CBX3, or CBX5) (Canzio et al., 2014) are interesting candidates as mediators of clique assembly. Physically, TAD clique formation could involve a phase separation process, which has been shown to be implicated in the formation of heterochromatin and in driving the segregation of heterochromatin from euchromatin (Larson et al., 2017; Strom et al., 2017). If TADs in a clique do not physically contact each other at the single-cell level, but are rather in a close neighborhood, proximity of TADs in cliques could be mediated by liquid condensates aggregating and constraining specific homotypic chromatin domains in a confined space (Shin et al., 2018). Supporting this idea are demonstrations of clustering of enhancers (Sabari et al., 2018) and formation of Polycomb condensates by phase separation (Tatavosian et al., 2019). The latter could potentially explain a subset of H3K27me3-rich TAD cliques in A compartments (Paulsen et al., 2019) and other Polycomb domains (Fraser et al., 2015; Olivares-Chauvet et al., 2016).

Loss-of-function experiments should provide clues on factors involved in the gain or loss of TADs in cliques. What is currently missing is a robust method to assay TAD clique formation or breakdown, which would not depend on costly and labor-intensive Hi-C experiments. High-throughput FISH assays (Finn et al., 2019), TAD visualization in living cells using CRISPR/Cas9-EGFP marking of domains (Wang and Qi, 2016) or using genetic tagging with the ANCHOR system (Bystricky, 2015; Germier et al., 2017), may be tools worthy of investigation to monitor TAD clique expansion, shrinking and spatial distribution in the nucleus. Live-cell chromatin imaging methods would also enable visualization of TAD clique dynamics and spatial tracking in real time.

Variability in spatial genome conformations highlighted in single-cell experiments raises the issue of to whether TAD clique dynamics represents a deterministic or stochastic process (Bystricky, 2015). The current lack of demonstration that TAD clique assembly and disassembly is a regulated process opens for possibilities that stochasticity plays a significant role in spatial genome configurations (Flyamer et al., 2017). Inasmuch as stochasticity in gene expression emerges as an important contributor to regulated gene expression patterns (Dessalles et al., 2017; Horowitz and Kulkarni, 2017), stochasticity in genome conformation may favor preferred topologies that direct gene expression programs. Such deterministic view of genome structure-function relationships at higher-order level remains to be examined in 4D contexts using matched topological and transcriptome datasets. Along these lines, more detailed analyses of the links between differentiation- and lineage-specific TAD clique formation and expression control of genes within TAD cliques will in the future help gaining further insights into the significant of these higher-order long-range domain associations.

Are TAD cliques deregulated in disease contexts? TAD cliques harbor a large number of disease-associated genes in normal cultured MSCs. Lamin A modulates large-scale chromatin dynamics (Bronshtein et al., 2015) and contributes to the peripheral anchoring of heterochromatin at the nuclear envelope (Solovei et al., 2013). So the role of A-type lamins in the regulation of TAD cliques, and whether they are differentially affected in B versus A compartments, will be important to investigate in the context of lamin A mutations causing laminopathies (Worman, 2012) – in particular mutations that affect lamin-chromatin interactions and gene positioning (Mewborn et al., 2010; Perovanovic et al., 2016; Paulsen et al., 2017; Briand and Collas, 2018; Briand et al., 2018). In the near future, the combination of strategies including high-throughput genome editing and genomics (Leemans et al., 2019), high-throughput FISH (Finn et al., 2019), live-cell imaging of chromatin (Germier et al., 2017), biophysical approaches and computational modeling methods will enhance our knowledge of the functional relationship between genome organizers in a 4D nucleome perspective.

## Author Contributions

All authors have contributed to the conception of this article and approved it for publication.

## Conflict of Interest Statement

The authors declare that the research was conducted in the absence of any commercial or financial relationships that could be construed as a potential conflict of interest.
